# G-CSF-induced ANCA associated glomerulonephritis in the presence of silent membranous “full house nephropathy” in an altruistic bone marrow donor

**DOI:** 10.1080/0886022X.2022.2115380

**Published:** 2022-08-24

**Authors:** Avital Angel Korman, Adi Leiba, Yonatan Edel, Vladimir Rapoport, Ana Tobar, Merav Leiba

**Affiliations:** a Nephrology and Hypertension Institute, Samson Assuta University Hospital, Ashdod, Israel; b Faculty of Health Sciences, Ben Gurion University of the Negev, Beer-Sheva, Israel; c Department of Internal Medicine ‘B’, Assuta Ashdod University Hospital, Ashdod, Israel; d Department of Pathology, Beilinson Medical Center, Petah Tikva, Israel; e Hematology Institute, Samson Assuta University Hospital, Ashdod, Israel

Dear Editor,

Neupogen (Filgrastim) is a recombinant human granulocyte colony stimulating factor (G-CSF) commonly used to stimulate progenitor cell collection for the purpose of bone marrow transplantation (BMT) [1].

G-CSF activates inflammatory cells including neutrophils, and leads to increased production of chemokines and inflammatory cytokines [2]. Filgrastim has also been connected to ANCA associated vasculitis, likely through its effect on neutrophils [3].

We present here a case of a seemingly healthy altruistic bone marrow (BM) donor who developed acute GN which was unmasked by the use of G-CSF.

A 34-year old healthy man was seen at our Nephrology clinic ten days following a 4-day course of the G-CSF Neupogen (Filgrastim), for the purpose of altruistic BM donation. Immediately after G-CSF treatment, he developed flu-like symptoms including fatigue, and headaches which were pursued by epistaxis, painless macrohematuria and AKI with elevation of his serum creatinine up to 2.9 mg/dL (baseline level 1.2–1.3 mg/dL).

On physical examination his blood pressure was 131/62–140/73 mmHg and the rest of the physical examination was unremarkable. He denied cigarette smoking, alcohol abuse or illicit drug use, but admitted taking three doses of ibuprofen (400 mg each) for pain relief.

Urinalysis demonstrated hematuria (250cells/microliter) and proteinuria (11–50mg/dL). A renal ultrasound demonstrated hyperechogenic, normal sized (12 cm) kidneys.

The patient was admitted to the hospital with a presumed flare of IgA nephropathy.

On further questioning, he reported a similar episode seven years prior, manifested as macrohematuria, AKI (creatinine elevated to −1.6 mg/dL) accompanied by headache and sinusitis without fever. Urinalysis at the time was positive for blood and 50 mg/dL of protein. Workup revealed a positive Anti Streptolysin O antibodies and normal complement levels, without clinical extra-renal signs of vasculitis. He was treated with doxycycline for presumed atypical infection. Creatinine level was re-checked 3 years later and was 1.17 mg/dL, presumed to represent his baseline level.

Of note, several urinalyses performed following his initial presentation with AKI, demonstrated persistent microhematuria up to 250 cells/microliter without proteinuria.

One year prior to his current presentation, he suffered another episode of fever and macrohematuria with a creatinine level of 1.34 mg/dL which was not re-checked.

Importantly, these details in his medical history were not revealed during the screening process for altruistic stem cell donation or prior to G-CSF treatment.

During this current admission, urine protein to creatinine ratio (UPCR) was 630 mg/gram and CRP was 4.0 mg/dL (normal range 0–0.5 mg/dL). Serological workup including complement levels (C3 and C4) and immunoglobulin levels were within normal range. RF, HIV, HBV, and HCV antibodies were unremarkable. Anti-proteinase-3, anti-glomerular basement membrane (GBM) and anti-nuclear antibody were all negative. Myeloperoxidase (MPO) antibodies were found to be elevated to 97 IU/mL (normal range 0–3.5 IU/mL).

**Figure 1.**
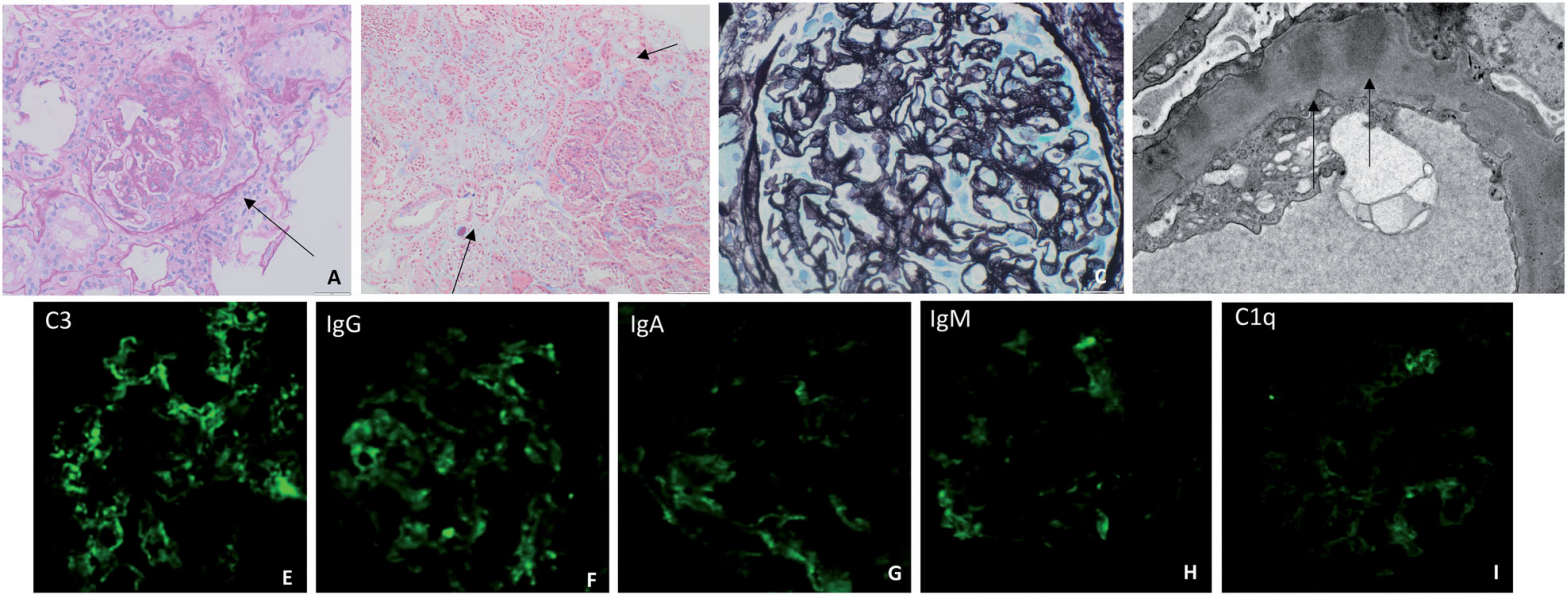
Kidney biopsy findings. (A) LM, PAS stain, glomerulus with a cellular crescent; (B) LM, Masson staining, intra tubular hematinic casts surrounded by interstitial inflammatory cells and mild fibrosis; (C) LM, Silver stain, thickened glomerular basement membranes with spikes; (D) EM, thickened glomerular basement membrane with intramembranous dense deposits; E-I. IF, C3, C1q and immunoglobulins.

A kidney biopsy was performed as shown in Figure 1.

On light microscopy 2/11 glomeruli were globally sclerosed, and one glomerulus contained a cellular crescent. Small fresh thrombi were seen in the lumen of scattered glomerular capillaries. There were GBM and mesangial thickening. Capillary loop ‘spikes’ were seen on silver staining. Mild interstitial inflammation and hematinic casts were present in the tubular lumen.

Immunofluorescence showed granular deposition of C3, C4, IgG, IgM, IgA and C1q along the GBM and mesangium, consistent with a ‘full house’ pattern. PLA2R was negative and no IgA or IgG subtype dominance was noted.

Electron Microscopy revealed extensive electron dense deposits, both subepithelial and intramembranous at different stages of reabsorption along with scattered mesangial and subendothelial deposits. There were no intense C1q staining, dominance of mesangial, subendothelial or tubular deposits, and no tubulo-reticular inclusions were seen to suggest that either the membranous or the ‘full house’ immunofluorescence patterns were related to lupus nephritis. The final diagnosis was therefore: acute focal crescentic GN on top of atypical membranous nephropathy with a full house pattern.

As we suspected filgrastim to be the trigger of his current flare, we avoided treatment with steroids or other immunosuppressive agents.

On a follow-up visit to the clinic 6 weeks later, creatinine declined to baseline levels (1.2–1.3 mg/dL), although with persistent microhematuria. Six months following filgrastim course, a urine creatinine clearance test demonstrated a measured GFR of 47 mL/min. Repeat UPCR was still elevated to 750 mg/g creatinine and anti MPO titer continued to be significantly increased to 128 IU/mL, with a stable serum creatinine, still under no immunosuppressive treatment.

Exacerbations of ANCA associated GN [3,4] have been connected with G-CSF treatment.

ANCA vasculitis is a neutrophil predominant vasculitis with myeloid dysregulation which drives endothelial injury [5]. G-CSF enhances neutrophil activation, which activates endothelial cells through the release of von-Willebrand factor and thrombomodulin. This results in E-selectin ligand expression enhancement, leading to attachment of neutrophils to the injured endothelium [6].

In this case the clinical scenario (previous AKI episodes, persistent microhematuria) along with kidney biopsy findings, are consistent with a previously undiscovered baseline glomerulopathy.

We argue that following G-CSF treatment, MPO antibodies were formed leading to signs and symptoms of ANCA vasculitis correlating to a Birmingham vasculitis activity score (BVAS) of 18 ‘new’ points. The activated neutrophils were attracted to the formerly deposited immunoglobulins and complement in the glomeruli, leading to the formation of a cellular (indicating fresh) crescent as well as fresh thrombi thus serving as a ‘second hit’. This cascade of events could have easily gone unnoticed if no baseline glomerulopathy had been present. The baseline glomerulopathy in this case – full house nephropathy (FHN) was the reason for the pathology findings on immunofluorescence, which were not consistent with pure ANCA vasculitis.

Five additional cases of GN developing on top of possible previous glomerulopathies following G-CSF administration, are described in Table 1. Nevertheless, the current case is the first report of this specific rare glomerulopathy – atypical membranous FHN, exacerbated by G-CSF exposure.

**Table 1. Cases of G-CSF induced glomerulonephritis on top of pre-existing glomerulopathy.** 

Patient characteristics	G-CSF induced disease (2nd hit)	Background Glomerulopathy (1st hit)	Reference
Healthy altruistic allogeneic BM donor	MPO- associated GN	Atypical full house membranous nephropathy	Current case
Patient with scleroderma after autologous stem cell transplantation	Acute IgA nephropathy	Intermittent transient hematuria	[7]
Autologous BM transplantation for Multiple Myeloma	Crescentic GN	proliferative GN with monoclonal IgG2λ deposits	[8]
BM donation to HLA matched brother with ALL	Acute IC mediated GN	Morbid obesity (BMI- 42), Hypertension, possible microscopic hematuria	[9]
endogenous overproduction of G-CSF	Membranoproliferative GN	Persistent Microhematuria	[10]
Allogeneic BM donor to father [4]	Pauci-immune MPO- associated GN	Protein and creatine supplements, mild proteinuria and microhematuria	[4]

Full House Nephropathy is an immune-complex mediated glomerulopathy, that resembles lupus nephritis in its ‘full house’ immunofluorescence pattern, however with no other (clinical, serological or pathological) features suggestive of SLE. FHN can be either idiopathic, which usually carries a grave renal prognosis, or secondary to other conditions, of which the most common is membranous nephropathy [11,12].

The clinical and histopathological features in our patient suggest the diagnosis of idiopathic non-lupus FHN with atypical membranous pattern. According to Rijnink et al. [12] treatment with immunosuppression (mainly including corticosteroids) did no alter the grave renal prognosis compared to those patients with non-lupus FHN that did not receive immunosuppressive therapy. We therefore continued to actively follow the patient without administration of immunosuppressive therapy.

To our knowledge, this is the first case described in an altruistic BM donor, who developed acute GN as a complication of G-CSF. Both this case and prior cases described in the literature seem to have in common a background glomerulopathy as a risk factor for G-CSF induced GN.

We therefore suggest that a workup, including a careful history oriented to glomerular pathologies, urinalysis, urine protein quantification and possibly a urine microscopy, be a routine part of the screening process for BM donors. Such a strategy can help identify patients at risk for developing G-CSF induced kidney injury, especially those with preexisting glomerulopathies.
